# Supplementary material for: Optimising non-invasive brain-computer interface systems for free communication between naïve human participants
Source: Sci Rep. 2019 Dec 10;9:18705. doi: 10.1038/s41598-019-55166-y (PMC6904487; doi:10.1038/s41598-019-55166-y)
Supplement: Supplementary file 1 — Supplementary Information [file 41598_2019_55166_MOESM1_ESM.pdf]

Optimising non-invasive brain-computer interface systems for free communication between naïve human participants

Supplementary Information

Angela I. Renton[1*], Jason B. Mattingley[1,2,3], & David R. Painter[2]

1. Queensland Brain Institute, The University of Queensland, St Lucia 4072, Australia
2. School of Psychology, The University of Queensland, St Lucia 4072, Australia
3. Canadian Institute for Advanced Research (CIFAR), Toronto, Canada

***Corresponding Author**

Angela I. Renton

Queensland Brain Institute

The University of Queensland

St Lucia, Queensland, 4072

Australia

Tel + 61 (7) 3346 6300

Email: angie.renton23@gmail.com

Appendix 1. Pool of prompt words for the free association task of Experiment 1.

ABLE	CHILDHOOD	FATHER	HOT	MAYBE	PLAY	SIMPLE	WHITE
ACTUALLY	CITY	FEAR	HOUR	MEAN	PLEASURE	SIMPLY	WHOLE
AHEAD	CLARITY	FEDERAL	HOUSE	MEDICAL	POINT	SINGLE	WILL
AIR	CLEAR	FEEL	HUGE	MEMBER	POLITICAL	SLEEP	WISDOM
ALONE	CLEARLY	FINAL	HUMAN	MIGHT	POOR	SMALL	WOMAN
ALREADY	CLOSE	FINALLY	IDEA	MILITARY	POPULAR	SOCIAL	WORD
AMERICAN	CLOSE	FINANCIAL	IMPORTANT	MINUTE	POSSIBLE	SOMETIMES	WORK
APPETITE	COLD	FIND	INDEED	MOMENT	POWER	SOON	WORK
AREA	COLDNESS	FINE	INFORMATION	MONEY	PRESIDENT	SORROW	WORLD
AROUND	COME	FORCE	INSTEAD	MONTH	PRETTY	SPECIAL	WOULD
ART	COMMON	FOREIGN	INTERNATIONAL	MORNING	PRIVATE	START	WRONG
ASK	COMMUNITY	FORWARD	ISSUE	MOTHER	PROBABLY	STATE	YEAR
AVAILABLE	COMPANY	FREE	JOB	MOVE	PROBLEM	STORY	YOUNG
AWARENESS	COULD	FREEDOM	JUSTICE	MOVEMENT	PROGRAM	STRONG	CHILD
BACK	COUNTRY	FRIEND	KEEP	MUST	PUBLIC	STUDENT	FAST
BAD	CULTURAL	FRIENDSHIP	KID	NAME	PUT	STUDY	HOME
BEAUTY	CURRENT	FULL	KIND	NATIONAL	QUESTION	STUPIDITY	MAY
BECOME	DARK	GAME	KNOW	NATURAL	QUICKLY	SUDDENLY	PLACE
BEFORE	DAY	GENERAL	LATE	NEARLY	QUITE	SURE	SIMILAR
BEGIN	DEAD	GENEROSITY	LAW	NEED	RATHER	SYSTEM	WEEK
BELIEF	DEMOCRATIC	GET	LEAVE	NEW	READY	TAKE	
BELIEVE	DIFFERENT	GIRL	LEFT	NICE	REAL	TALK	
BEST	DIFFICULT	GIVE	LEGAL	NIGHT	REASON	TEACHER	
BEST	DIRECTLY	GOOD	LESS	NO	RECENT	TEAM	
BETTER	DOOR	GOODNESS	LET	NUMBER	RECENTLY	TELL	
BETTER	EARLY	GOVERNMENT	LEVEL	OF COURSE	RED	THAT	
BIG	EARLY	GREAT	LIFE	OFFICE	RESEARCH	THING	
BLACK	EASY	GROUP	LIKE	OK	RESULT	THINK	
BODY	ECONOMIC	GUY	LIKELY	OLD	RIGHT	THUS	
BOOK	EDUCATION	HAND	LINE	ONLY	RIGHT	TIME	
BOTH	EITHER	HAPPEN	LITTLE	OPEN	RIGHT	TOGETHER	
BRAVERY	ELSE	HAPPINESS	LIVE	OTHER	ROOM	TONIGHT	
BRING	END	HAPPY	LOCAL	OTHERS	RUN	TRADITIONAL	
BUSINESS	ENOUGH	HARD	LONG	PARENT	SCHOOL	TRU	
CALL	ENTIRE	HARD	LOOK	PART	SEE	TRY	
CALM	ENVIRONMENTAL	HEAD	LOT	PARTICULARLY	SEEM	TURN	
CAN	ESPECIALLY	HEALTH	LOW	PARTY	SERIOUS	USE	
CAR	EVENTUALLY	HEAR	LUCK	PAST	SERVICE	USUALLY	
CASE	EXACTLY	HELP	LUXURY	PEOPLE	SHORT	VARIOUS	
CENTRAL	EYE	HIGH	MAIN	PERHAPS	SHOULD	WANT	
CERTAIN	FACE	HISTORY	MAJOR	PERSON	SHOW	WAR	
CERTAINLY	FACT	HOLD	MAKE	PERSONAL	SIDE	WATER	
CHANGE	FAMILY	HOME	MAN	PHYSICAL	SIGNIFICANT	WAY	

Appendix 2. Freely associated and BCI typed words and phrases in Experiment 1.

Accuracy indicates whether the BCI typed word/phrase matched (1) or mismatched (0) the text manually entered using a physical keyboard, which provided the ground truth for communication intent.

Cue Word	BCI Typed Word/Phrase	Accuracy
ABLE	ABLE IS A GOOD SONG BY THE NATIONAL	1
ABLE	TO	1
ABLE	TO	1
ACTUALLY	DONT YOU ACTUALLY ME	1
AIR	BREATH	1
APPETITE	HUNGRY	1
AREA	LOCATION	1
AROUND	NEARBY	1
AROUND	THE BLOCK	1
ART	PAINTING	1
ASK	PERMISSION	1
AVAILABLE	WIFI	1
AVAILABLE	SINGLE	1
BACK	FRONT	1
BACK	PLANE	1
BAD	GOOD	1
BAD	GOOD	1
BEAUTY	UGLY	1
BECOME	A PRINCESS	1
BEFORE	AFTER	1
BEGIN	ALL OVER AGAIN YOUVE GOT TO BE KIDDING	1
BEGIN	END	1
BELIEVE	TRUST	1
BEST	DAY OF MY LIFE	1
BETTER	GOOD	1
BLACK	NIGHT	1
BOOK	LIBRARY	1
CALL	ME BABY	1
CALL	CENTRE	1
CALM	CALM	1
CAN	BE	1
CAR	ACCIDENT	1
CASE	IS ONE LETTER AWAY FROM CHASE	1
CERTAIN	CERTAIN	1
CHANGE	REMAIN	1
CHILD	TOY	1
CLARITY	HEAL	1

CLEAR	CRYSTAL	1
CLEAR	GLAS	1
CLEARLY	NOT	1
CLOSE	OPEN	1
COLDNESS	HEATER	1
COME	ARRIVE	1
COMMON	SENSE	1
COMMON	SENSE	1
COMMON	SENSE	1
COMMUNITY	FRIENDS	1
COMMUNITY	COLLEGE	1
COMPANY	FUN	1
COUNTRY	FLAG	1
CULTURAL	MUSEUM	1
CURRENT	VOLTAGE	1
DAY	NIGHT	1
DEAD	I WANT ONE OF THESE ON MY PHONE	1
DEAD	DEAD HARD	1
DEAD	TREE	1
DEAD	OR ALIVE	1
DEMOCRATIC	COUNTRY	1
DEMOCRATIC	LIBERAL	1
DIFFERENT	SAME	1
DIFFICULT	TASK	1
DIRECTLY	INCOME	1
DIRECTLY	SENT	1
DIRECTLY	INDIRECTLY	1
DOOR	HAW PAR VILLA	1
EARLY	BLUE	1
EARLY	LATE	1
EARLY	PROSOPAGNOSIA	1
EASY	EASY DOES IT	1
EASY	HARD	1
EASY	DIFFICULT	1
EDUCATION	SYSTEM	1
END	OF THE DAY	1
END	FINAL	1
END	HOLIDAY	1
END	START	1
ENTIRE	ENTIRE LIFE	1
ENVIRONMENTAL	IVE GOT NOTHING	1
ENVIRONMENTAL	ECOLOGICAL	1
EVENTUALLY	NIGHT	1
EVENTUALLY	FINALLY	1
EVENTUALLY	TOMORROW	1
EYE	TEST	1

EYE	FOR AN EYE	1
FATHER	MOTHER	1
FEAR	PHOBIA	1
FEDERAL	TAX EVASION	1
FEEL	THE MOMENT	1
FINALLY	YOURE TELLING ME	1
FIND	OUT	1
FIND	SENTOSA	1
FINE	DINING	1
FINE	ROUGH	1
FOREIGN	FROM BACK HERE	1
FORWARD	BACKWARD	1
FORWARD	EFFECT	1
FREE	FREEDOM	1
FREE	EXPENSIVE	1
FREEDOM	SET	1
GAME	GAMEBOY	1
GENERAL	BLACK	1
GENERAL	GENERAL STUDIES	1
GENEROSITY	PROSPERITY	1
GET	HAVE	1
GOODNESS	GOOD	1
GOODNESS	GOD	1
GOODNESS	OH MY	1
GOVERNMENT	PINK DOT	1
GREAT	AWESOME	1
GREAT	DIM SUM	1
GROUP	WORK	1
GUY	GIRL	1
HAPPEN	SHIT	1
HAPPINESS	WEDDING	1
HARD	WORK	1
HEAD	SHOULDER	1
HEALTH	GOOD	1
HEAR	MUSIC	1
HISTORY	WILLIAM WILLIAM HENRY STEVEN	1
HISTORY	EDUCATION	1
HOLD	WAIT	1
HOT	DOG	1
HOT	TEA	1
HOUR	LATE	1
HOUR	SECOND	1
HUMAN	BEING	1
HUMAN	BEING	1
HUMAN	BEING	1
INFORMATION	INFORMATION CENTRE	1

INSTEAD	OF THAT ILL DO THIS	1
INSTEAD	CONTRARY	1
INSTEAD	OF	1
INTERNATIONAL	FLIGHT	1
INTERNATIONAL	STUDENT	1
ISSUE	FINANCIAL	1
JOB	SUIT	1
JOB	INTERVIEW	1
JUSTICE	FAIR	1
JUSTICE	METALLICA	1
JUSTICE	FAIR	1
JUSTICE	FREEDOM	1
KEEP	KEEP GOING	1
KIND	NICE	1
LAW	AND ORDER SVU	1
LEGAL	LAWYERS	1
LESS	FOOD	1
LET	IT BE	1
LET	GO	1
LEVEL	UP	1
LEVEL	FLOOR	1
LEVEL	THREE	1
LIKE	HATE	1
LINE	APP	1
LINE	DOTTED	1
LONG	HOURS	1
LOOK	UP	1
LOOK	ZEBRA	1
LOT	OF	1
LOW	KEY	1
LUCK	FORTUNE	1
LUCK	NO PAU	1
LUCK	UNLUCKY	1
MAKE	MUFFINS	1
MEAN	DO YOU MEAN NASTY OR AVERAGE	1
MEAN	KIND	1
MEDICAL	SCIENCE	1
MEDICAL	IMAGING	1
MEMBER	SHIP	1
MIGHT	NIGHT	1
MIGHT	HAVE	1
MILITARY	TRAINING	1
MINUTE	HOUR	1
MOMENT	NOW	1
MOMENT	SUPPER	1
MONEY	CASH	1

MONTH	MONTHLY	1
MORNING	AFTERNOON	1
MOTHER	FATHER	1
MOVE	GYM	1
MOVE	HOUSE	1
MOVE	STAY	1
MOVEMENT	STEADY	1
MUST	IS NOT A WORD I PARTICULARLY LIKE	1
NATIONAL	CONFERENCE	1
NATIONAL	MAGAZINE	1
NEED	USE	1
NEED	FOOD	1
NEW	OLD	1
NICE	GUY	1
NO	YES	1
OF	FOR	1
OFFICE	CHAIR	1
OK	AGREED	1
OK	ADVENTURE	1
ONLY	ONE	1
ONLY	YOU	1
ONLY	ONE	1
OTHER	THAN YOU	1
OTHER	OTTERS	1
OTHER	PEOPLE	1
OTHERS	FAULT	1
PARENT	CHILD	1
PARENT	CHILDREN	1
PART	QUARTER	1
PARTICULARLY	PIZZA	1
PEOPLE	ANIMAL	1
PERHAPS	PERHAPS YOU	1
PERSON	INDIVIDUAL	1
PERSON	ANIMAL	1
PLACE	IT PUTS THE LOTION ON ITS SKIN	1
PLACE	HOUSE	1
PLAY	GROUND	1
PLAY	TIME	1
POOR	RICH	1
POOR	RICH	1
POPULAR	FAMOUS	1
PRESIDENT	OBAMA	1
PRESIDENT	POLITIC	1
PRESIDENT	DRUMPF	1
PRETTY	UGLY	1
PROBABLY	NOT	1

PROBABLY	NOT	1
PROGRAM	COURSE	1
PROGRAM	ROBOT	1
PUBLIC	HOSPITAL	1
PUT	IN THE BOX	1
QUESTION	ANSWER	1
RATHER	WOULD	1
REASON	EXCUSE	1
REASON	HABITAT	1
RECENTLY	IM SURE WE DID THINGS ON THE WEEKEND	1
RED	BLOODSHOT	1
RESEARCH	SCIENCE	1
RESULT	EXAM	1
RIGHT	NOT	1
RIGHT	WRONG	1
SEE	EYES	1
SEEM	S TO BE	1
SEEM	TO BE	1
SERIOUS	GAME	1
SERIOUS	JOKING	1
SERVICE	GOOD	1
SERVICE	FEE	1
SERVICE	OCCUPATION	1
SHORT	IS THE NICKNAME MY DAD GAVE ME AS A CHILD	1
SHOULD	BE CORRECT	1
SIDE	BY SIDE	1
SIDE	BY SIDE	1
SIMILAR	IS DIFFERENT TO DIFFERENT	1
SIMILAR	SAME	1
SINGLE	COUPLE	1
SINGLE	DOUBLE	1
SLEEP	TIME	1
SMALL	BIG	1
SMALL	LARGE	1
SOCIAL	SCIENCE	1
SOMETIMES	I GET HUNGRY	1
SOON	HOME	1
SORROW	TEARS	1
SPECIAL	TYPE	1
SPECIAL	THINGS	1
START	STARTER	1
STATE	OF ORIGIN	1
STORY	BED TIME	1
STRONG	WEAK	1
STUPIDITY	SMART	1
SUDDENLY	GONE	1

SYSTEM	WOW	1
TALK	CHAT	1
TEACHER	BLACKBOARD	1
THING	THINK	1
THINK	LETS GIVE THIS A TRY	1
THINK	CAP	1
THUS	SO	1
THUS	THUS YOU	1
TIME	IS UP	1
TOGETHER	ALONE	1
TOGETHER	FRIENDS	1
TONIGHT	BED	1
TRADITIONAL	ANCIENT	1
TRUE	FALSE	1
TRY	I AM TRYING FUCK YOU	1
TRY	WALL	1
TURN	QWERTY BACKWARDS IS YTREWQ	1
TURN	LEFT	1
USE	USEFUL	1
USUALLY	USUALLY I USE MY FINGERS FOR THIS	1
USUALLY	REMIND	1
VARIOUS	THINGS	1
VARIOUS	REASONS	1
WAR	WEAPON	1
WATER	BOTTLE	1
WAY	OUT	1
WAY	PATH	1
WAY	HEY	1
WILL	JUST LEFT	1
WORD	BALL	1
WORK	BUSY AND STRESSFUL BUT FUN	1
WORK	SLACKING	1
WORLD	DOMINATION	1
WOULD	COULD	1
YOUNG	WILD	1
YOUNG	TEENAGER	1
ALONE	LONW	0
AROUND	ROX	0
AROUND	AROUND YOH	0
AVAILABLE	OFFLIWG	0
BAD	BAE	0
BEAUTY	WOMKH	0
BEFORE	AFT	0
BETTER	WOR	0
CLOSE	DOOF	0
COME	ONO	0

COULD	BL	0
DEMOCRATIC	FR	0
EARLY	EAGLY STAGEUN	0
ECONOMIC	XYLOP	0
ELSE	R	0
ENOUGH	IS ENOUGHGTWAX	0
FEDERAL	COU	0
FEEL	FECC	0
FINALLY	EVENTUALLYY	0
FORWARD	BAC KS	0
FRIENDSHIP	A	0
FULL	GLASSF	0
GIVE	F	0
GOVERNMENT	VOTM	0
HAND	LITL	0
HAPPEN	HAPPINEXF	0
HAPPY	SMILJ	0
HARD	EARUET	0
HISTORY	SUBJY	0
HUGE	ENORMYMO	0
IMPORTANT	MIIDO	0
JOB	JOB	0
KIND	KINU	0
LESS	MO	0
LIKELY	THIEUOX	0
LIVE		0
LONG	FLIGITQB	0
MILITARY	ARFO	0
NATURAL	BORN HIPFHQB	0
NICE	JOURNEKWT	0
OF	OF COURSV	0
PART	PKGWYFAPFXQAYQLGRZKJABYOPPBUAI	0
PART	PART TIMWEU	0
PAST	PAST TENTS	0
PEOPLE	ALOIMB	0
PERHAPS	E	0
PERHAPS	MAY	0
PERSON	PERSONALRAU	0
PRIVATE	SECRE	0
PRIVATE	SECSNB	0
PUBLIC	PUBLIC TRANSPO	0
QUITE	QUIFI	0
QUITE	CONTRA	0
RECENTLY	BYXBKYPPT	0
RUN	RA	0
SIMPLY	SIMP	0

SINGLE	SINGLE LA	0
STUDENT	STUDEN	0
TEAM	FOTGT	0
TELL	SAY	0
THAT	THAT RIGHYAL	0
TURN	TURN	0
WEEK	NIGHT SAFA	0
WHITE	BLACE	0
WORD	LO	0
WORK	HARO	0
YOUNG	OLRXM	0

Appendix 3. Transcript of the free communication of Experiment 2.

Note that all characters in names have been replaced with dashes ("-") to protect participant identities.

P2: HUNGRY
P1: HALLO
P2: AS A HIPPO
P1: ALSO HUNGRY
P2: WIE GETP
P1: NOT A HIPPO
P2: TYPEYTYPETYPE
P1: MIR GEHT ES GUT
P2: HALO ----
P1: HI -----
P2: HOW WAS YOUR WE
P2: WKEND
P1: MY WEEKEND WAS GREAT WENT TO THE BEACH YOURS
P2: R U DOING ANYTHING FOR HALLOWEEN
P1: NOT SURE YET DO YOU
P2: ALSO GOOD DID TRAPEZE
P2: SO MUCH FUN
P2: LIKE FLYING
P2: YEAH WENT TO A FRENCH PARTY
P1: DRP WE NEED A QKLSMGY
P1: QUEST
P2: -------- WAS ATROPHY WIFE
P1: QUESTIONMARK
P2: HER COSTUME WAS AMAZIN
P1: THAT IS A GREAT COSTUME
P1: WHAT DID YOU WEAR
P2: YEAH SHE HAD PEDASTAL AND EVERYTHING
P1: I WOULD DRESS UP AS A SHRUB
P2: THEME WAS GOLD SO PAINTED SELF GOLD
P1: EASY
P2: HAHAHAHE
P2: HOW
P1: ARE U GOING TO -----S
P2: YEAH
P2: PLS COME AS A SHRUB
P2: SPECIFICALLY AS MONTY PYTHON SHRUBBERY
P1: BUY A BIG POT AND STEAL SHRUB MATERIAL FROM NEIGHBOUR
P1: ILL TRY
P1: DO U HAVE A COSTUME
P2: THIS SPELLER MAKES ME VERY AWARE OF HOW SHODDY MY SPELLING IS
P1: AND I JUST LEARNT A NEW WORD SHODDY
P2: GOING AS THE UNICORN FROM HRRY PTTR THAT GETS EATEN BY VOLDEMORT
P1: IS IT A WORD OR YOUR SHODDY SPELLING
P2: ITS A WORD EXCLAMATION POINT
P1: BRING SPARKLY GIN AS UNICORN BLOOD
P1: GOOD TO KNOW ILL TRUST YOU
P2: OR MY PARENTS MADE IT UP TO AVOID SWEARING IN FRONTOF ME AS A KID
P2: OOOH GENIUS
P1: QUITE POSSIBLE THAT IS WHAT PARENTS DO

P2: I DIDNT KNOW THERE WAS SPARKLY GIN
P2: HOWS YOUR TYPING GOING
P1: ITS PROBABLY TOXIC BUT FOR A SHORT MOMENT OF YOUR LIFE YOU WILL FEEL LIKE A UNICORN
P2: I THINK MY GEL IS DRYING A LITTLE
P1: PRETTY SWEET
P1: TYPING WAS NEVER BETTER
P1: ENTERTAING THE READERS WHILE YOU ARE REFRESHING YOUR GEL
P2: IM GETTING SOME COLOURED CONTACTS TO COMMIT FULLY HAVE ALWAYS WANTED TO TRY THEM
P1: ARE U BACK HELLO HELLO
P2: HELLO AGAIN
P1: HAHA GOOD LUCK GETTING USED TO THEM
P2: DO YOU LIKE HOUSE PLANTS
P1: YOU WILL SPEND THE NIGHT CRYING
P2: WAS TALKING TO - ----- TODAY
P1: I LIKE ALL PLANTS
P2: HES CLEARING OUT HIS GARDEN AND BRINGING IN A BUNCH OF CUTTINGS
P2: I GUESS TIMES UP BYE ----
P1: TALKED TO HIM ABOUT HALLUCINATING CATS NOT PLANTS
